# Insights From the Analysis of Clinicopathological and Prognostic Factors in Patients With Gallbladder Cancer

**DOI:** 10.3389/fonc.2022.889334

**Published:** 2022-04-14

**Authors:** Di Wu, Wenjian Jin, Yue Zhang, Yong An, Xuemin Chen, Weibo Chen

**Affiliations:** Department of Hepatopancreatobiliary, Third Affiliated Hospital of Soochow University, Changzhou, China

**Keywords:** gallbladder cancer (GBC), preventive surgery, advanced gallbladder cancer, comprehensive therapy, prognostic factors

## Abstract

**Aims:**

To investigate the clinical efficacy and prognostic factors of primary gallbladder cancer (GBC) treated by radical surgery.

**Methods:**

The clinical and pathological data of 168 patients with primary gallbladder cancer admitted and treated in the Third Affiliated Hospital of Soochow University from January 1st, 2010 to December 31st, 2018 were analyzed retrospectively. Kaplan Meier method was used to draw the survival curve and evaluate the survival rate. Chi-square test was used for univariate analysis and binary logistic regression was used for multivariate analysis.

**Results:**

94 cases showed symptoms of abdominal pain and abdominal distension. 7 cases showed symptoms of fatigue and weight loss. Jaundice occurred in 10 patients. Fever occurred in 6 patients. 51 patients had no symptoms at all. The median survival time of 168 patients was 35.0 (1.0 ~ 142.0) months. The overall 1-, 2- and 3-year cumulative survival rates were 69.6%, 55.4% and 48.8% respectively. The univariate analysis indicated that preoperative bilirubin, tumor size, tumor location, pathological type, degree of differentiation, liver invasion, nerve invasion, vascular invasion, surgical margin, filtration depth and N staging were significant factors influencing prognosis of patients with primary GBC (P<0.05). The results of multivariate analysis demonstrated that degree of differentiation, nerve invasion, filtration depth and N staging were independent risk factors for prognosis of patients with primary GBC (P<0.05).

**Conclusion:**

Patients with risk factors of gallbladder cancer should be more active in early cholecystectomy to avoid the malignant transformation of benign diseases. Degree of differentiation, nerve invasion, filtration depth and N staging were important factors for poor prognosis of patients with primary GBC. For T4 staging patients, preoperative evaluation should be more comprehensive, and patients and surgeons should be more prudent in adopting appropriate clinical treatment. The primary purpose should be prolonging the survival time and improving the quality of life.

## Introduction

Primary GBC is one of the most common malignant tumors in the biliary system. GBC is highly malignant, the early diagnosis of which is difficult, and the prognosis is very poor. Radical resection is still the most effective treatment to improve the prognosis of GBC patients (1). The 5-year survival rate of gallbladder cancer is only about 10% ~ 30% (2). In recent years, with the deepening understanding of the disease, its treatment mode has been transformed into multidisciplinary comprehensive treatment based on surgery. The scope of liver resection and lymph node dissection has been expanded. Adjuvant treatments such as postoperative chemotherapy, preoperative neoadjuvant chemotherapy and targeted drug therapy have been gradually carried out (3). Even so, the long-term survival rate is still poor. Therefore, this study retrospectively analyzed the clinical and pathological data of 168 patients with GBC treated in the Third Affiliated Hospital of Soochow University from January 1st, 2010 to December 31st, 2018 to explore the long-term clinical efficacy of radical resection and independent risk factors affecting the prognosis of patients with GBC.

## Materials and Methods

### Study Design and Data Collection

This study is a retrospective comparative study performed in a single center. Data were collected retrospectively.

### Inclusion Criteria and Exclusion Criteria

The inclusion criteria were as follows: 1. Postoperative routine pathology confirmed the diagnosis of GBC, 2. No distant metastasis occurred, 3. Patients all underwent radical surgery, 4. No other malignant tumors existed.

The exclusion criteria were as follows: 1. Patients underwent palliative surgery or patients underwent preoperative neoadjuvant therapy, 2. Clinical data of the patients were incomplete, or the patients were lost during follow-up.

### Preoperative Examination

Laboratory tests include blood routine test, hepatic and renal functions, tumor markers and so on. Electrocardiogram and pulmonary function were routinely performed to exclude the surgical contraindications. B ultrasound, CT and (or) MRI were performed to obtain a correct diagnosis. PET-CT was used when previous examination could not provide a clear diagnose or distant metastasis could not be excluded.

### Surgical Procedure

Preoperative staging and operation plan was made according to preoperative examination. The stages were further defined by surgical exploration and rapid pathology, and the corresponding radical surgery was carried out based on different stages. Patients under stage tis and T1a underwent laparoscopic or open cholecystectomy while patients under T1b~T3 needed cholecystectomy + lymph node dissection + hepatectomy according to different conditions. (For T1b and T2 staging patients, wedge resection of liver was performed, and for T3 staging patients, resection of segment IVB+V of liver was performed). However, for patients under stage T4, combined organ resections such as right colectomy, subtotal gastrectomy and pancreatoduodenectomy were needed. Extent of lymph-node dissection included dissection of group 8, 12 and 13a lymph nodes.

### Pathological Diagnostic Criteria

Tumor staging was evaluated based on the 8th edition of AJCC Cancer staging system.

### Clinical and Pathological Materials

The clinical manifestations, preoperative imaging and laboratory examination results, surgical methods, pathological data, and survival time were analyzed. Gender, age, preoperative total bilirubin, tumor size, tumor location, pathological type, degree of differentiation, liver invasion, nerve invasion, vascular invasion, surgical margin, filtration depth, N stage and postoperative adjuvant chemotherapy were included in univariate and multivariate analysis.

### Follow-Up Model

All patients were followed up by telephone or by out-patient. The follow up period ended on December 31, 2021, or death.

### Statistical Analysis

Normal distributions of numerical variable data were verified with Shapiro Wilk test. Numerical variable data were presented as mean and standard deviation (SD). Categorical variable data were presented as number and percentages (4). Kaplan Meier method was used to draw the survival curve and calculate the survival rate. χ 2 test and Fisher’s exact test was used for univariate survival analysis, and Cox regression model was used for multivariate analysis. SPSS software (SPSS Statistics 17.0) was used for data analysis and a statistically significant difference was considered for a value of P<0.05.

## Results

### General Information of Patients

A total of 168 patients with primary gallbladder cancer were included, including 52 males and 116 females, with a male to female ratio of 0.45:1. The age ranged from 29 to 90 years old, with an average of (65.14 ± 11.78) years old.

### Clinical Manifestation

94 cases showed symptoms of abdominal pain and abdominal distension. 7 cases showed symptoms of fatigue and weight loss. Jaundice occurred in 10 patients. Fever occurred in 6 patients. 51 patients had no symptoms at all.

### Diagnostic Imaging and Laboratory Data

The diagnostic accuracy of abdominal B-ultrasound, CT and MRI were 48.34% (73/151), 73.60% (92/125), and 59.67% (37/62), while the diagnostic accuracy of CEA, CA199, CA125 were 26.67% (36/135), 45.45% (60/132), and 20.77% (27/130).

### Surgical Procedure

All the 168 patients underwent radical resection of GBC. Among them, 41 patients underwent laparoscopic surgery, including 13 cases of laparoscopic cholecystectomy and 28 cases of laparoscopic radical resection. The rest 127 patients underwent open surgery, among which, 117 patients underwent wedge resection or IVB+V segment resection of liver. The rest included 4 cases of hepatopancreaticoduodenectomy (HPD), 3 cases of right hepatectomy and 3 cases of combined subtotal gastrectomy and right colectomy.

### Postoperative Complications

Postoperative complications occurred in ten patients. Postoperative bleeding was observed in three patients, but none of them needed second operation. All they needed were prolonged drain time and intravenous infusion of hemostatic drugs. Two patients developed pleural effusion and were relieved after pleural puncture and drainage. Two patients developed bile leakage and three patients developed pancreatic fistula and they were improved by prolonged drain time.

### Pathological Results

153 patients were diagnosed with adenocarcinoma. Among them, there were 3 cases of papillary adenocarcinoma, 3 cases of tubular adenocarcinoma, 1 case of fungating adenocarcinoma. Non adenocarcinoma patients include 11 cases of adeno-squamous carcinoma, 3 cases of squamous cell carcinoma and 1 case of sarcomatoid carcinoma. As with the degree of differentiation, 80 patients were highly differentiated, 45 patients were moderately differentiated, and 43 patients were poorly differentiated. In 86 cases, the tumors were located at the bottom of the gallbladder, 62 at the body of the gallbladder and 20 at the neck of the gallbladder. Liver invasion occurred in 50 patients. Nerve invasion occurred in 28 patients. Vascular invasion occurred in 18 patients. The number of patients with tumor stage 0, I, II, III, IV were 9, 26, 57, 62 and 14 respectively.

### Follow-Up Data

**Figure 1:**
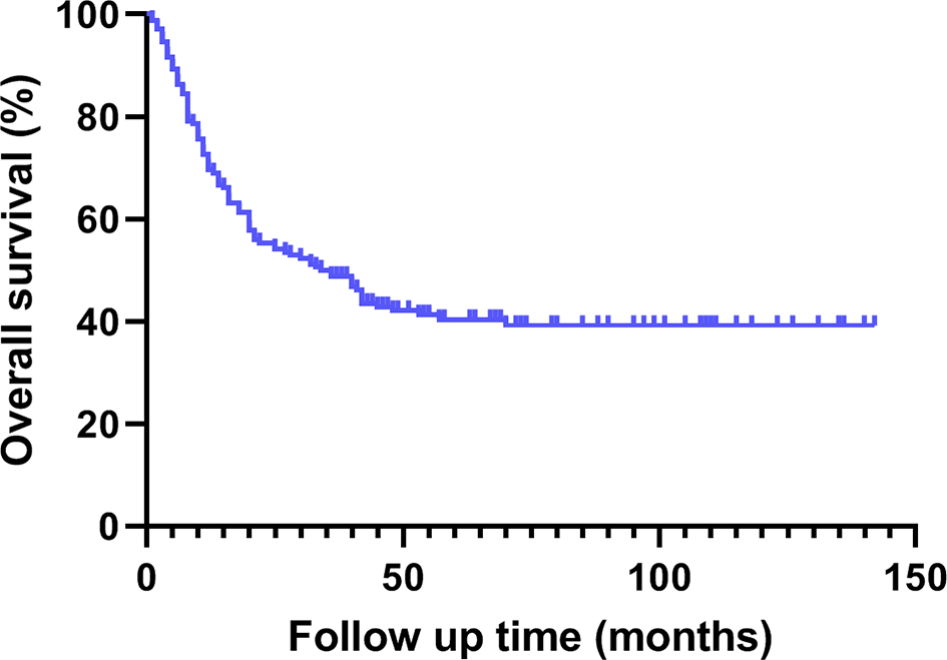
Overall survival curve of 168 patients with gallbladder cancer treated by radical surgery.

**Figure 2:**
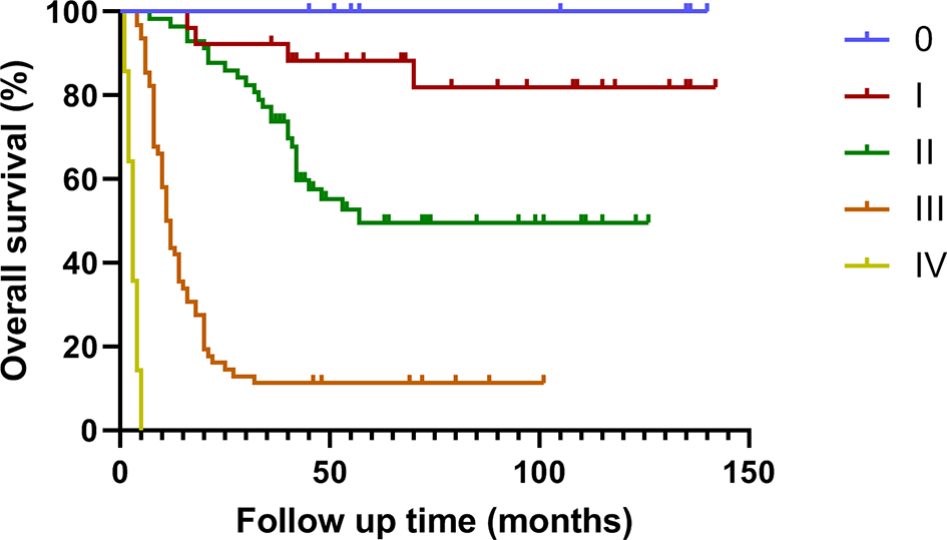
Survival curve of patients with different stages of gallbladder cancer treated by radical surgery.

The median survival time of this group was 35.0 (1.0 ~ 142.0) months. The cumulative survival rates of all patients at 1, 2 and 3 years were 69.6%, 55.4% and 48.8% respectively (**Figures 1**, **2**).

### Univariate Analysis of Prognosis

Univariate analysis showed that preoperative total bilirubin, tumor size, tumor location, pathological type, degree of differentiation, liver invasion, nerve invasion, vascular invasion, surgical margin, filtration depth and N stage were the risk factors affecting the prognosis of gallbladder cancer (P < 0.05). Gender, age, and postoperative adjuvant chemotherapy were not important factors affecting the prognosis (**Table 1**).

**Table 1:** Univariate prognostic analysis of 168 patients with gallbladder cancer.

Clinicopathological factors	Number of patients	Three-year survival rate	Chi square value	p Value
Sex			1.782	0.243
Male	52	57.7		
Female	116	46.6		
Age (Years)			0.033	0.877
<65	70	51.4		
≥65	98	50		
Total bilirubin (umol/L)			7.01	0.009
<100	158	53.2		
<100	10	10		
Tumor size (cm)			9.08	0.009
<2cm	22	72.7		
2~5cm	119	51.3		
>5cm	27	29.6		
Tumor location			12.59	0.001
Gallbladder bottom	86	47.7		
Gallbladder body	62	64.5		
Gallbladder neck	20	20		
Pathological type			6.665	0.013
Adenocarcinoma	153	54.9		
Non adenocarcinoma	15	20		
Degree of differentiation			75.565	0.000
High differentiation	80	83.8		
Medium differentiation	45	35.6		
Low differentiation	43	4.7		
Liver invasion			56.636	0.000
No	118	69.5		
Yes	50	6		
Nerve invasion			25.38	0.000
No	140	59.3		
Yes	28	7.1		
Vascular invasion			4.199	0.048
No	150	53.3		
Yes	18	27.8		
Surgical margin			5.052	0.022
Negative	149	53.7		
Positive	19	26.3		
Filtration depth			93.247	0.000
Tis~T2	104	79.8		
T3	54	3.7		
T4	10	0		
N staging			56.707	0.000
N0(*)	113	70.8		
N1	46	10.9		
N2	9	0		
Postoperative adjuvant chemotherapy			0.233	0.682
Yes	28	46.4		
No	140	51.4		

^*^The pathological results of 18 cases were either carcinoma in situ or tumors confined to mucosa, therefore, lymph node dissection wasn’t conducted.

### Multivariate Analysis of Prognostic Factors

Multivariate analysis demonstrated that degree of differentiation, nerve invasion, filtration depth and N staging were important factors for poor prognosis of patients with primary GBC (P < 0.05) (**Table 2**).

**Table 2:** Multivariate prognostic analysis of 168 patients with gallbladder cancer.

Clinicopathological factors	B value	Standard error	Wald value	OR	95%CI	P value
Degree of differentiation	1.654	0.417	15.753	5.230	2.311~11.840	0.000
Nerve invasion	3.211	1.017	9.970	24.808	3.380~182.086	0.002
Filtration depth	3.964	0.859	21.314	52.642	9.785~283.202	0.000
N staging	1.772	0.738	5.761	5.880	1.384~24.982	0.016

## Discussion

Gallbladder cancer is a highly malignant tumor of the digestive system. It has no special manifestations at an early stage. When non-specific symptoms such as upper abdominal pain, abdominal distension, and even jaundice occurred, it often indicated tumors in progressive stage. The incidence rate of gallbladder cancer in China accounts for 0.4%~3.8% of biliary tract diseases, ranking sixth in digestive tract cancer, and 5 years survival rate of gallbladder cancer is only 5% (5). Risk factors for gallbladder cancer consist of cholecystolithiasis, polypoid lesions of the gallbladder and chronic cholecystitis. In our study, 51 patients had no symptoms at all. They were diagnosed by B-ultrasound, CT, or MRI by accident. Most of the 51 patients had risk factors for gallbladder cancer. None of them had underwent preventive cholecystectomy before malignant transformation.

It is reported that about 85% of patients with gallbladder cancer are complicated with cholecystolithiasis (5). The risk of gallbladder cancer in patients with cholecystolithiasis is 13.7 times higher than that in people without cholecystolithiasis. The diameter and number of gallstones are positively correlated with the occurrence of gallbladder cancer. Cholesterol and mixed cholesterol gallstones have an even higher risk (6).

Cholecystectomy is recommended for symptomatic cholecystolithiasis (7). For asymptomatic cholecystolithiasis, whether surgical treatment should be performed is still controversial in China. As far as I am concerned, since gallstone is a risk factor for gallbladder cancer, gallstone is one of the surgical indications. But right now, for Chinese people, it is unacceptable to resect a “normal” organ without any symptoms. Patients with asymptomatic cholecystolithiasis who are not willing to undergo surgery for the time being should be closely followed up. Cholecystectomy should be carried out in time in case of clinical symptoms occurred, or cholecystolithiasis related complications (acute pancreatitis, common bile duct stones or cholangitis, etc.) occurred or when there were risk factors for gallbladder cancer (7, 8).

Multivariate analysis demonstrated that the filtration depth is one of the risk factors affecting the prognosis of gallbladder cancer, which is consistent with the latest research results (9–11). In our study, the median survival time of T4 patients after extended radical resection was 3 months, which was much lower than that of other stages. It is still controversial whether T4 stage gallbladder cancer without distant metastasis can be treated with extended radical resection combined with multiorgan resection and vascular reconstruction. A retrospective clinical study by Chen et al. showed that the median survival time of stage T4 gallbladder cancer without surgery was only 2.3 months, while the survival time could be significantly prolonged by radical surgery (12). D’Souza insisted that although HPD was associated with perioperative mortality and morbidity, it can still offer a survival benefit in patients with GBC (13). Kuipers had reached the similar conclusion (14). However, some experts took a more cautious attitude. Considering the high morbidity rate and the advanced stage of the disease, Sakamoto hold the idea that the indication for HPD in advanced stage gallbladder cancer should be considered carefully (15). In our study, 4 patients underwent HPD, and three of them had developed pancreatic fistula. In our opinion, extended radical resection is a double-edged sword. Extended radical resection does provide a survival benefit. Perioperative mortality and morbidity can’t be neglected. HPD carries high morbidity and mortality rates. The reported incidence of mortality after HPD has been reported to be as high as 33% (16–19). Hepatic failure and pancreatic fistula account for most of the perioperative mortality. However, 3 patients undergoing right hepatectomy and 3 patients undergoing combined subtotal gastrectomy and right colectomy, no postoperative complications occurred. Few studies have reported the perioperative mortality and morbidity of extended radical resection other than HPD. Therefore, for extended radical resection other than HPD, adequate preoperative evaluation including general condition, liver function, nutritional status should be accomplished. As with HPD, treatment decisions should be more conservative. After all, the primary purpose of medical treatment should be prolonging the survival time and improving the quality of life. We still have many options dealing with GBC besides surgery. In recent years, many clinical studies have explored the adjuvant chemotherapy, which has changed the treatment status of gallbladder cancer (20–24). At the same time, molecular targeted therapy and immunotherapy of gallbladder cancer have also achieved encouraging results. In the future, comprehensive therapy is expected to bring greater survival benefits to patients with gallbladder cancer.

Still, our work has its limitations. First of all, it was performed in a single center. The results may be influenced by subjective factors such as surgical skills. Secondly, this study was a retrospective study and prospective randomized controlled trial is more convincing. In the near future, maybe we will participate in a multicenter randomized controlled trial.

In conclusion, patients with risk factors of gallbladder cancer should be more active in early cholecystectomy to avoid the malignant transformation of benign diseases. For T4 staging patients, preoperative evaluation should be more comprehensive, and patients and surgeons should be more prudent in adopting appropriate clinical treatment. The primary purpose should be prolonging the survival time and improving the quality of life.

## Data Availability Statement

The raw data supporting the conclusions of this article will be made available by the authors, without undue reservation.

## Ethics Statement

The studies involving human participants were reviewed and approved by Third Affiliated Hospital of Soochow University. The patients/participants provided their written informed consent to participate in this study.

## Author Contributions

WC and XC designed the research and revised the manuscript. XC and YZ carried out the surgery with the help of their colleagues. YA and WJ gathered results of each patient. WJ analyzed the data. DW wrote the manuscript and took an active part in the procedures mentioned above. All authors contributed to the article and approved the submitted version.

## Funding

This study was supported by Young Talent Development Plan of Changzhou Health Commission (No. CZQM2021002, No. CZQM2020005), Young talent science and technology project of Changzhou Health Commission (No. QN202101) and the National Natural Science Foundation of China (No. 81602054).

## Conflict of Interest

The authors declare that the research was conducted in the absence of any commercial or financial relationships that could be construed as a potential conflict of interest.

## Publisher’s Note

All claims expressed in this article are solely those of the authors and do not necessarily represent those of their affiliated organizations, or those of the publisher, the editors and the reviewers. Any product that may be evaluated in this article, or claim that may be made by its manufacturer, is not guaranteed or endorsed by the publisher.

## Abbreviations

GBC, gallbladder cancer; CT, computed tomography; MRI, magnetic resonance imaging; PET-CT, positron emission tomography computed tomography; AJCC, American joint committee on cancer; SD, standard deviation; CEA, carcinoembryonic antigen; CA199, carbohydrate antigen 199; CA125, carbohydrate antigen 125; HPD, hepatopancreaticoduodenectomy.

## References

[1] Wistuba II Gazdar AF. Gallbladder Cancer: Lessons From a Rare Tumour. Nat Rev Cancer (2004) 4(9):695–706. doi: 10.1038/nrc1429 15343276

[2] Miranda-Filho A Pineros M Ferreccio C Adsay V Soerjomataram I Bray F. Gallbladder and Extrahepatic Bile Duct Cancers in the Americas: Incidence and Mortality Patterns and Trends. Int J Cancer (2020) 147(4):978–89. doi: 10.1002/ijc.32863 PMC8629410 31922259

[3] Lindner P Holmberg E Hafstrom L. Gallbladder Cancer - No Improvement in Survival Over Time in a Swedish Population. Acta Oncol (2018) 57(11):1482–9. doi: 10.1080/0284186X.2018.1478124 29932778

[4] Tan X Wang G Tang Y Bai J Tao K Ye L. Minilaparoscopic Versus Single Incision Cholecystectomy for the Treatment of Cholecystolithiasis: A Meta-Analysis and Systematic Review. BMC Surg (2017) 17(1):91. doi: 10.1186/s12893-017-0287-x 28830403 PMC5568361

[5] Hundal R Shaffer EA. Gallbladder Cancer: Epidemiology and Outcome. Clin Epidemiol (2014) 6:99–109. doi: 10.2147/CLEP.S37357 24634588 PMC3952897

[6] Roa I Ibacache G Roa J Araya J de Aretxabala X Munoz S. Gallstones and Gallbladder Cancer-Volume and Weight of Gallstones are Associated With Gallbladder Cancer: A Case-Control Study. J Surg Oncol (2006) 93(8):624–8. doi: 10.1002/jso.20528 16724353

[7] Gutt C Schlafer S Lammert F. The Treatment of Gallstone Disease. Dtsch Arztebl Int (2020) 117(9):148–58. doi: 10.3238/arztebl.2020.0148 PMC7132079 32234195

[8] Tazuma S Unno M Igarashi Y Inui K Uchiyama K Kai M. Evidence-Based Clinical Practice Guidelines for Cholelithiasis 2016. J Gastroenterol (2017) 52(3):276–300. doi: 10.1007/s00535-016-1289-7 27942871

[9] Yang XW Chen JY Wen ZJ Li YL Wang FY Li L. Effect of Preoperative Jaundice on Long-Term Prognosis of Gallbladder Carcinoma With Radical Resection. World J Surg Oncol (2020) 18(1):239. doi: 10.1186/s12957-020-02015-2 32891147 PMC7487893

[10] Gani F Buettner S Margonis GA Ethun CG Poultsides G Tran T. Assessing the Impact of Common Bile Duct Resection in the Surgical Management of Gallbladder Cancer. J Surg Oncol (2016) 114(2):176–80. doi: 10.1002/jso.24283 PMC5450028 27198742

[11] Higuchi R Yazawa T Uemura S Matsunaga Y Ota T Araida T. Examination of Prognostic Factors Affecting Long-Term Survival of Patients With Stage 3/4 Gallbladder Cancer Without Distant Metastasis. Cancers (Basel) (2020) 12(8):2073. doi: 10.3390/cancers12082073 PMC7464443 32726993

[12] Chen C Geng Z Shen H Song H Zhao Y Zhang G. Long-Term Outcomes and Prognostic Factors in Advanced Gallbladder Cancer: Focus on the Advanced T Stage. PloS One (2016) 11(11):e0166361. doi: 10.1371/journal.pone.0166361 27846279 PMC5112857

[13] D’Souza MA Valdimarsson VT Campagnaro T Cauchy F Chatzizacharias NA D’Hondt M. Hepatopancreatoduodenectomy -a Controversial Treatment for Bile Duct and Gallbladder Cancer From a European Perspective. HPB (Oxford) (2020) 22(9):1339–48. doi: 10.1016/j.hpb.2019.12.008 31899044

[14] Kuipers H de Savornin Lohman EAJ van Dooren M Braat AE Daams F van Dam R. Extended Resections for Advanced Gallbladder Cancer: Results From a Nationwide Cohort Study. Ann Surg Oncol (2021) 28(2):835–43. doi: 10.1245/s10434-020-08858-z PMC7801314 32696306

[15] Sakamoto Y Nara S Kishi Y Esaki M Shimada K Kokudo N. Is Extended Hemihepatectomy Plus Pancreaticoduodenectomy Justified for Advanced Bile Duct Cancer and Gallbladder Cancer? Surgery (2013) 153(6):794–800. doi: 10.1016/j.surg.2012.11.024 23415082

[16] Nimura Y Hayakawa N Kamiya J Maeda S Kondo S Yasui A. Hepatopancreatoduodenectomy for Advanced Carcinoma of the Biliary Tract. Hepatogastroenterology (1991) 38(2):170–5. doi: 10.1136/gut.32.4.458-a 1649788

[17] Tsukada K Yoshida K Aono T Koyama S Shirai Y Uchida K. Major Hepatectomy and Pancreatoduodenectomy for Advanced Carcinoma of the Biliary Tract. Br J Surg (1994) 81(1):108–10. doi: 10.1002/bjs.1800810139 7906181

[18] Nakamura S Nishiyama R Yokoi Y Serizawa A Nishiwaki Y Konno H. Hepatopancreatoduodenectomy for Advanced Gallbladder Carcinoma. Arch Surg (1994) 129(6):625–9. doi: 10.1001/archsurg.1994.01420300069010 7515618

[19] Miyagawa S Makuuchi M Kawasaki S Hayashi K Harada H Kitamura H. Outcome of Major Hepatectomy With Pancreatoduodenectomy for Advanced Biliary Malignancies. World J Surg (1996) 20(1):77–80. doi: 10.1007/s002689900014 8588418

[20] Mizusawa J Morizane C Okusaka T Katayama H Ishii H Fukuda H. Randomized Phase III Study of Gemcitabine Plus s-1 Versus Gemcitabine Plus Cisplatin in Advanced Biliary Tract Cancer: Japan Clinical Oncology Group Study (JCOG1113, FUGA-BT). Jpn J Clin Oncol (2016) 46(4):385–8. doi: 10.1093/jjco/hyv213 PMC4886137 27025903

[21] Ben-Josef E Guthrie KA El-Khoueiry AB Corless CL Zalupski MM Lowy AM. SWOG S0809: A Phase II Intergroup Trial of Adjuvant Capecitabine and Gemcitabine Followed by Radiotherapy and Concurrent Capecitabine in Extrahepatic Cholangiocarcinoma and Gallbladder Carcinoma. J Clin Oncol (2015) 33(24):2617–22. doi: 10.1200/JCO.2014.60.2219 PMC4534524 25964250

[22] Fukutomi A Furuse J Okusaka T Miyazaki M Taketsuna M Koshiji M. Effect of Biliary Drainage on Chemotherapy in Patients With Biliary Tract Cancer: An Exploratory Analysis of the BT22 Study. HPB (Oxford) (2012) 14(4):221–7. doi: 10.1111/j.1477-2574.2011.00431.x PMC3371207 22404259

[23] Edeline J Benabdelghani M Bertaut A Watelet J Hammel P Joly JP. Gemcitabine and Oxaliplatin Chemotherapy or Surveillance in Resected Biliary Tract Cancer (PRODIGE 12-ACCORD 18-UNICANCER GI): A Randomized Phase III Study. J Clin Oncol (2019) 37(8):658–67. doi: 10.1200/JCO.18.00050 30707660

[24] Primrose JN Fox RP Palmer DH Malik HZ Prasad R Mirza D. Capecitabine Compared With Observation in Resected Biliary Tract Cancer (BILCAP): A Randomised, Controlled, Multicentre, Phase 3 Study. Lancet Oncol (2019) 20(5):663–73. doi: 10.1016/S1470-2045(18)30915-X 30922733

